# Microbial gene expression during hibernation in arctic ground squirrels: greater differences across gut sections than in response to pre-hibernation dietary protein content

**DOI:** 10.3389/fgene.2023.1210143

**Published:** 2023-08-10

**Authors:** Kirsten Grond, C. Loren Buck, Khrystyne N. Duddleston

**Affiliations:** ^1^ Department of Biological Sciences, University of Alaska Anchorage, Anchorage, AK, United States; ^2^ Department of Biological Sciences, Northern Arizona University, Flagstaff, AZ, United States

**Keywords:** hibernation, urea nitrogen salvage, metatranscriptome analysis, dietary protein, arctic ground squirrel, urease, amino acids (AA)

## Abstract

Obligate seasonal hibernators fast for 5–9 months depending on species yet resist muscle atrophy and emerge with little lean mass loss. The role of the gut microbiome in host nitrogen metabolism during hibernation is therefore of considerable interest, and recent studies support a role for urea nitrogen salvage (UNS) in host-protein conservation. We were interested in the effect of pre-hibernation diet on UNS and the microbial provision of essential amino acids (EAAs) during hibernation; therefore, we conducted a study whereby we fed arctic ground squirrels (*Urocitellus parryii*) pre-hibernation diets containing 9% vs. 18% protein and compared the expression of gut bacterial urease and amino acid (AA) metabolism genes in 4 gut sections (cecum mucosa, cecum lumen, small intestine [SI] mucosa, and SI lumen) during hibernation. We found that pre-hibernation dietary protein content did not affect expression of complete bacterial AA pathway genes during hibernation; however, several individual genes within EAA pathways were differentially expressed in squirrels fed 18% pre-hibernation dietary protein. Expression of genes associated with AA pathways was highest in the SI and lowest in the cecum mucosa. Additionally, the SI was the dominant expression site of AA and urease genes and was distinct from other sections in its overall microbial functional and taxonomic composition. Urease expression in the gut microbiome of hibernating squirrels significantly differed by gut section, but not by pre-hibernation dietary protein content. We identified two individual genes that are part of the urea cycle and involved in arginine biosynthesis, which were significantly more highly expressed in the cecum lumen and SI mucosa of squirrels fed a pre-hibernation diet containing 18% protein. Six bacterial genera were responsible for 99% of urease gene expression: *Cupriavidus*, *Burkholderia*, *Laribacter*, *Bradhyrizobium*, *Helicobacter*, and *Yersinia.* Although we did not find a strong effect of pre-hibernation dietary protein content on urease or AA metabolism gene expression during hibernation, our data do suggest the potential for pre-hibernation diet to modulate gut microbiota function during hibernation, and further investigations are warranted.

## Introduction

The host-gut microbiome relationship is one in which the host provides an anaerobic environment and access to dietary substrates, and the gut microbiome provides myriad ecosystem services such as immune regulation, detoxification, and nutrient absorption (Huttenhower et al., 2012; Brestoff and Artis, 2013; Kostic et al., 2013). Most gut microbes rely on dietary components that escape intestinal absorption and, to a lesser extent, endogenous host substrates (e.g., shed epithelial cells and mucin glycans); thus, the macronutrient content of host diet is a major factor that regulates gut microbial community structure and function (Sonnenburg et al., 2005; Scott et al., 2013; Beam et al., 2021).

Microbe-facilitated gut processes can affect host energy and nutrient metabolism by providing the host with essential nutrients such as vitamins and short chain fatty acids (SCFA), and contributing to nutrient homeostasis (Nogal et al., 2021). For example, nitrogen is a main component of proteins, nucleic acids, and other biomolecules, and maintaining a balanced nitrogen metabolism is essential (Waterlow, 1999). In mammals, nitrogen is acquired through the diet and used in biosynthesis of nitrogen-containing biological macromolecules such as proteins. Many amino acids (AAs) are synthesized by the liver (i.e., the “non-essential amino acids”; NEAAs); however, the essential amino acids (EAAs) must be acquired from the diet, or supplied by gut bacteria that synthesize EAAs using either dietary or host-recycled nitrogen (e.g., urea) (Hou et al., 2015; Newsome et al., 2020).

**FIGURE 1:**
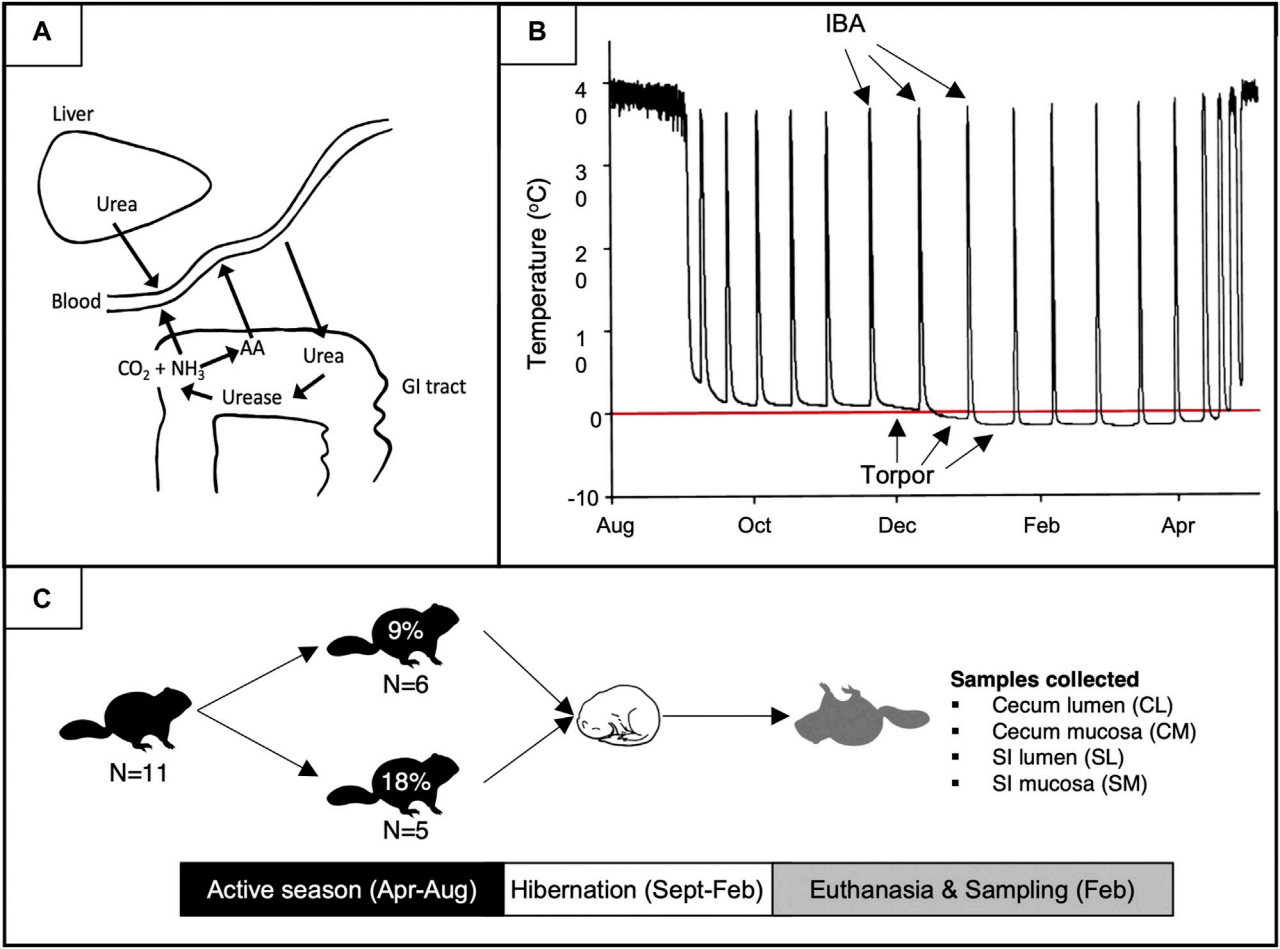
Schematic of UNS processes **(A)**, hibernation **(B)**, and the experimental design of this study **(C)**. (A) The dynamic between torpor bouts and interbout arousals (IBAs). The red line depicts 0°C. (B) Urea-nitrogen-salvage and urea-derived nitrogen use in host amino acid (AA) formation. (C) Experimental design and sample collection. Percentages represent the % dietary protein fed to squirrels during the pre-hibernation season. Figure A was adapted from Stewart and Smith (2005).

The use of host-recycled nitrogen occurs via urea-nitrogen-salvage (UNS), a process whereby urea produced by the liver diffuses into the gut and is hydrolyzed to ammonia and carbon dioxide by urease-containing gut bacteria (Figure 1B; Stewart and Smith, 2005). The liberated urea-nitrogen may subsequently be reabsorbed as ammonia and used by the host, or it may be utilized by gut microbes to produce AAs and other nitrogen-containing biomolecules that are subsequently taken up by the host. Host recycling of microbially-liberated urea-nitrogen has been identified across most animal tax including humans. Indeed, mammals, birds, amphibians, and fish have all been shown to use UNS as a nitrogen conserving mechanism (Singer, 2003; Stewart and Smith, 2005; Wiebler et al., 2018). In addition, dietary protein content can influence the extent to which hosts rely on UNS for nitrogen conservation. For example, humans consuming a low protein diet salvaged 64% of nitrogen in host-produced urea compared to 46% of those consuming high protein diets (Langran, 1992), and our preliminary data indicate that arctic ground squirrels incorporate more microbially-liberated urea-nitrogen into their tissues when consuming 9% compared to 18% dietary protein (unpublished data). In combination with studies demonstrating the presence of EAAs of microbial origin in plasma and tissues of a diversity of mammals including humans (Torrallardona et al., 1996; Metges et al., 1999), these studies suggest the use of recycled nitrogen by gut bacteria to synthesize and supply EAAs could be especially important to host animals during times of low food availability or when host organisms consume a low protein diet.

Hibernation in mammals evolved to survive unfavorable environmental conditions. In obligate seasonal hibernators such as ground squirrels, hibernation is characterized by a prolonged state of inactivity and profoundly reduced metabolic rate and body temperature (T_b_) called torpor, bouts of which are periodically interrupted by < 24 h periods of euthermia and normal metabolic rate called interbout arousals (IBA). During hibernation, ground squirrels neither eat nor drink; rather, they subsist upon endogenous lipid reserves accrued during their active season. The arctic ground squirrel (*Urocitellus parryii*), considered the most extreme hibernator, prepares for hibernation by increasing body fat from 5% to 45% or more in as little as 3 weeks (Buck and Barnes, 1999a, b), and their hibernation season lasts up to 9 months (Buck and Barnes, 1999a; Sheriff et al., 2011). During hibernation bouts of torpor extend up to 25 days (Buck et al., 2008) during which Tb is regulated as low as −2.9°C (Barnes, 1989; Buck and Barnes, 2000; Richter et al., 2015). When arctic ground squirrels hibernate at ambient temperatures (T_a_) between 0°C and 8°C they rely solely on lipid metabolism (Buck and Barnes, 2000); however, as T_a_ decreases below 0°C, they switch from exclusively lipid metabolism to mixed fuel metabolism (Buck and Barnes, 2000) and are the only hibernating species known to do so.

Investigations of the gut microbiotas of hibernators have revealed that gut microbes are metabolically active during the interbout euthermic periods within the hibernation season (Stevenson et al., 2014), and that hibernation alters the diversity and composition of gut microbial communities (Carey et al., 2013; Stevenson et al., 2014; Tang et al., 2019; Xiao et al., 2019; Kurtz et al., 2020). Fasting during hibernation shifts the available nutrient pool for microbes to endogenous substrates such as mucins and sloughed epithelial cells, and selects for a gut microbiota enriched in mucin-degrading taxa (Carey et al., 2013; Stevenson et al., 2014).

The lack of dietary nitrogen during hibernation makes recycling of nitrogen *via* UNS critical for hibernators. For example, the American black bear (*Ursus americanus*) completely recycles urea-N to maintain lean body mass across their approximately 5-month hibernation season (Ahlquist et al., 1984; Harlow, H. 2012), and a recent study confirmed the occurrence of gut microbiome-mediated urea nitrogen recycling in hibernating thirteen-lined ground squirrels (*Ictidomys tridecemlineatus*; Regan et al., 2022). As noted previously, arctic ground squirrels shift from lipid to mixed-fuel metabolism when hibernating at T_a_s below 0°C (Buck and Barnes, 2000). They also upregulate protein synthesis and remodel specific tissues during hibernation (Lee et al., 2012; Fedorov et al., 2014), preserving lean mass, which suggests UNS and microbiota production of EAAs are likely important to meet their nitrogen needs.

We undertook a comprehensive study with the overarching goal of examining UNS and microbial provision of EAAs in hibernating arctic ground squirrels, and to assess the effect of pre-hibernation diet on the potential for UNS and microbial EAA production to meet nitrogen needs. We report here on a component of that overarching study in which we aimed to determine the effect of pre-hibernation dietary protein content on bacterial gene expression during hibernation in four different gut sections in the arctic ground squirrel: the cecum lumen, cecum mucosa, small intestine (SI) lumen, and SI mucosa. We focused on general metabolism pathways and AA-related pathways of bacteria with specific interest in processes involved in urea and EAA metabolism. Overall, we predicted that host pre-hibernation dietary protein content would modulate the genetic potential of the gut microbiota to contribute to UNS and bacterial AA metabolism during hibernation. Specifically, we hypothesized that squirrels on a low protein pre-hibernation diet would show higher expression of urease encoding genes during hibernation compared to those fed a high protein diet. We further predicted that squirrels on a low protein diet to show higher expression of bacterial genes associated with the biosynthesis of essential and non-essential amino acids.

## Methods

### Animal husbandry

Juvenile arctic ground squirrels were trapped in summer 2019 in the northern foothills of the Brooks Range, Alaska, near the Atigun River (688 38’ N; 1498 38’ W) using Tomahawk live traps baited with carrot. Squirrels were transported to the animal facility at the University of Alaska Anchorage within 7 days of trapping and quarantined for 2 weeks. Following quarantine, squirrels were surgically implanted with temperature-sensitive transmitters (TA10TA-F40; Data Sciences International [DSI], Holliston MA) to enable continuous monitoring of hibernation in real time (Barnes, 1989; Long et al., 2007).

Squirrels were housed individually in steel, hanging wire-bottom cages (18 ¾” W x 12 ¼” L x 12 ¼” D) at 20°C on a 12 h light schedule and provided with raw cotton bedding for nesting material, a metal tube for enrichment, and standard rodent chow (Mazuri, St. Louis, MO) with the addition of ∼5 g of apples daily, and water *ad libitum*. At the end of the first summer in captivity, squirrels were transferred to Nalgene^™^ tubs (15” W x 22” L x 8” D) with wood shavings, cotton nest material and steel wire lids. They were held in the dark in an environmental chamber at an ambient temperature (T_a_) of 2°C with neither food nor water and allowed to hibernate. In addition to electronic monitoring of hibernation stage via DSI temperature transmitters, squirrels were checked daily for signs of arousal using the sawdust method (Pengelley and Fisher, 1961).

## Experimental design

Following hibernation, yearling squirrels were transferred to hanging wire cages, held under euthermic conditions as described above, and divided into two dietary treatment groups fed a diet with either 9% (8 males +10 females; Teklad diet TD.170,165, Envigo [now Inotiv]) or 18% (7 males +6 females; Teklad diet TD.170164) protein content (Figure 1). Squirrels were fed ∼45 g of food daily and food was formed into a mush by adding water and 1 mL of sunflower oil to prevent caching and ensure all food was consumed. Squirrels received ∼5 g of apples daily for enrichment, and water was provided *ad libitum*. Squirrels were kept on these diets and weighed weekly for the duration of the active season until allowed to re-enter hibernation. Hibernating squirrels were housed as described above except at T_a_ = −16°C (T_a_ known to induce reliance on mixed-fuel metabolism in arctic ground squirrels; Buck and Barnes, 2000).

As noted above, the squirrels used in this study were part of a larger experiment examining UNS and microbial provision of EAAs. As part of the overarching project we used tracer metabolomics to examine the effect of pre-hibernation dietary protein content on the incorporation of microbially-liberated urea-nitrogen into gut microbial metabolites and host tissues during hibernation; thus, the experimental design included urea injections as follows: After at least 90 days of cumulative torpor, exclusive of IBAs, and within 2 days of the next predicted natural IBA, squirrels were transferred to a warm room (20°C), immediately given an intraperitoneal injection of either triply-labeled (^13^C^15^N^15^N 50 mg/kg; 99% ^13^C, 98% ^15^N_2_, Cambridge Isotope Laboratories, Inc; MA, United States) or unlabeled urea (Thermo Fisher Scientific, Waltham, MA), and artificially aroused. IBA timing was predicted by determining the average torpor bout length for each squirrel, which is known to be consistent across hibernation (Buck and Barnes, 2000; Buck et al., 2008). Squirrels were artificially aroused by keeping them in the warm room for approximately 5 h, and gently manipulating their limbs and rubbing their abdomens. After the squirrels were fully aroused, as determined by them actively moving around their hibernation tub, they were returned to the hibernation chamber to complete the IBA and re-enter torpor. A second injection of urea was administered within 2 days of the next predicted IBA and squirrels artificially aroused and then returned to the hibernation chamber following the identical protocol as for injection 1. After the squirrels had fully returned to torpor (∼24 h after the second injection) they were euthanized by decapitation for collection of samples. Squirrels were weighed at each injection and at euthanasia.

We collected and stored host tissue samples and gut contents from squirrels for tracer metabolomics, and gut contents for metagenomics. The methods and results of those studies are being reported under separate cover. We also collected gut contents from squirrels (see below) for the metatranscriptomics study we report here. All animal handling and research protocols were approved by the UAA Institutional Animal Care and Use Committee (1428231) and annual permits issued by the Alaska Department of Fish and Game (19-122, 20-108, 21-094).

### Sample collection

Squirrels were dissected immediately following euthanasia. For this study we collected both the lumen contents and the mucosa from the cecum and SI of hibernating squirrels that were fed 18% and 9% dietary protein during pre-hibernation. Cecal lumen contents were collected by removing the cecum and gently squeezing the lumen material into a sterile Petri dish. Then, to collect the cecal mucosa, the cecum was opened, gently rinsed with sterile saline, and the sample collected by scraping the inside of the cecum with a glass slide. The SI was also removed, and lumen samples collected by squeezing the material into a sterile Petri dish, after which we dissected the SI lengthwise, gently rinsed the tissue with sterile saline, and scraped the mucosa out with a sterilized spatula. Samples were transferred to 2 mL cryotubes and snap frozen in liquid nitrogen prior to storage at −80°C.

### Extraction and sequencing

We extracted RNA using the RNeasy PowerMicrobiome Extraction kit following manufacturer’s instructions (Qiagen, Hilden, Germany), and extracts were stored at −80°C until sequencing. Total RNA was sequenced by Genewiz (now Anta, South Plainfield, NJ). At the sequencing facility, the following protocol was used.

RNA samples were quantified using Qubit 2.0 Fluorometer (ThermoFisher Scientific, Waltham, MA, United States) and RNA integrity was checked with 4200 TapeStation (Agilent Technologies, Palo Alto, CA, United States). RNA samples were treated with TURBO DNase (Thermo Fisher Scientific, Waltham, MA, United States) to remove DNA following manufacturer’s protocol. rRNA depletion sequencing libraries were prepared by using QIAGEN FastSelect rRNA HMR + Bacteria Kit (Qiagen, Hilden, Germany). RNA sequencing library preparation uses NEBNext Ultra II RNA Library Preparation Kit for Illumina by following the manufacturer’s recommendations (NEB, Ipswich, MA, United States). Briefly, enriched RNAs are fragmented for 15 min at 94°C. First strand and second strand cDNA are subsequently synthesized. cDNA fragments are end repaired and adenylated at 3′ends, and universal adapters are ligated to cDNA fragments, followed by index addition and library enrichment with limited cycle PCR. Sequencing libraries were validated using the Agilent Tapestation 4200 (Agilent Technologies, Palo Alto, CA, United States), and quantified using Qubit 2.0 Fluorometer (ThermoFisher Scientific, Waltham, MA, United States) as well as by quantitative PCR (KAPA Biosystems, Wilmington, MA, United States). The sequencing libraries were multiplexed and clustered onto a flow cell. After clustering, the flow cell was loaded onto the Illumina NextSeq according to the manufacturer’s instructions. The samples were sequenced using a 2 × 150 bp Paired End (PE) configuration. Image analysis and base calling were conducted by the Control Software. Raw sequence data (.bcl files) generated from Illumina was converted into fastq files and de-multiplexed using Illumina bcl2fastq 2.20 software. One mis-match was allowed for index sequence identification.

### Sequence analysis

Sequence analysis was conducted following Grond et al. (2023). In short, sequences were trimmed using Trimmomatic using default parameters. Trimmed reads were *de-novo* assembled using Trinity (v. 2.6.6.) with a minimum contig length of 75 bp, and reads were separated into rRNA and other RNA using Sortmerna (v. 2.1 b). Non-rRNA reads were aligned using Bowtie2 and reads were annotated using Trinotate (Bryant et al., 2017; http://trinotate.github.io) against NCBI BlastX/P databases. Prior to statistical analysis, reads identified as eukaryotic were removed from the dataset.

### Statistical analysis

All statistical analyses were conducted in R (v.4.0.3). Prior to downstream analysis, expression counts were TMM (Trimmed Mean of M-values) normalized using the EdgeR package (v.3.40.2; Robinson et al., 2010). Principal Components Analysis was used on the TMM normalized expression counts significance and we calculated relative contributions of diet and gut subsection to functional composition of the gut bacterial communities. All Principal Components Analyses were based on correlation matrices. The effects of diet, gut section, and sex on functional and taxonomic community composition were visualized using principal component analyses (PCA), and the relative contribution of each variable to the variation on communities was calculated using Permutational multivariate analysis of variance (PERMANOVA) using the adonis function in vegan (v.2.6-4; Oksanen et al., 2022).

Analysis of Variance (ANOVA) was used to compare squirrel weights at time of injection/sampling and between sexes. Prior to these analyses, a Levene’s test was used to test for homogeneity of variance between sample groups. If heterogeneity in variance was detected, a Welch’s ANOVA was used instead.

Differential gene expression analysis between diets and gut sections was conducted using DESeq2 (v.1.38.3; Love et al., 2014) with significance set to a = 0.01. Volcano plots were constructed from DESeq data using EnhancedVolcano (v.1.16.0; Blighe et al., 2022) and other visualizations were made with ggplot2 (v.3.4.1; Wickham, 2016).

We were specifically interested in expression of bacterial genes involved in UNS, and for our analysis we focused on the urea cycle, urease gene expression, and AA metabolism. Within AA metabolism pathways, we investigated the biosynthesis and degradation of several EAAs and NEAAs. We identified genes coding for general metabolism pathways and genes that were involved in the urea cycle. To identify the urea-degradation genes involved in UNS and the physical location where UNS occurs in the squirrel gut, we also investigated genes associated with the urease operon. From urease genes detected, we determined the bacterial taxonomic origin to the genus and species level and visualized the topmost abundant genera comprising >99% of reads.

## Results

During the active season, squirrels fed an 18% protein diet gained weight faster than squirrels on a 9% protein diet (Supplementary Figure S1); however, weight gain converged between diets late in the season closer to the start of hibernation. The mass of squirrels was not affected by pre-hibernation diet at times of injection and euthanasia (Supplementary Figure S2; ANOVA: F_2,87_ = 0.114, *p* = 0.893). Male squirrels were significantly heavier than females regardless of pre-hibernation diet at time of injections and euthanasia (Supplementary Figure S3; ANOVA: F_1,88_ = 12.792, *p* < 0.001), but no differences were observed within females (ANOVA: *p* = F_1,45_ = 0.502, *p* = 0.609) or males (ANOVA: *p* = F_2,39_ = 0.088, *p* = 0.916). Average torpor bout length, IBA length, and body temperature during torpor and IBA did not differ between squirrels fed a 9% or 18% protein content pre-hibernation diet (Table 1. ANOVA: *p* = 0.197-0.682). We did not detect differences between sexes in the bacterial functional or taxonomic results presented below and therefore combined data from sexes to increase sample size.

**TABLE 1:** Hibernation characteristics of arctic ground squirrels used in our study. All data shown was collected prior to the first injection.

Dietary protein content (%)	Average torpor length (Days ±SE)	Average IBA length (hrs)^a^	Average torpor Tb (°C ± SE)	Average IBA Tb (°C ± SE)^b^
18	8.3 ± 0.8	10.8 ± 0.1	−1.5 ± 0.15	35.2 ± 0.12
9	8.5 ± 0.4	10.3 ± 0.3	−1.3 ± 0.15	34.4 ± 0.11

^a^ IBA, length defined as time above 30°C.

^b^ Maximum temperature during IBA.

### Function and taxonomy

**FIGURE 2:**
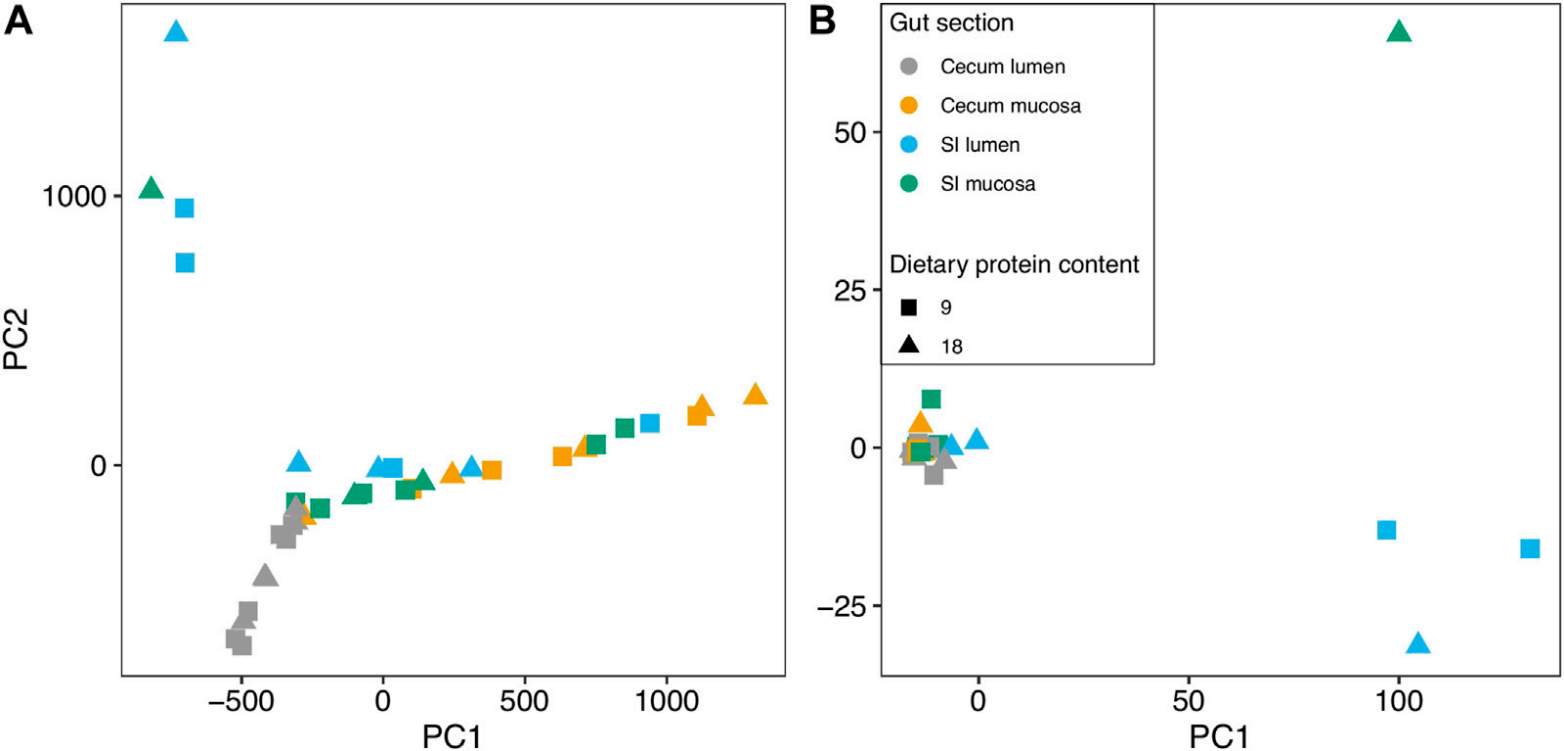
Principal Components Analysis of the **(A)** functional and **(B)** taxonomic community composition in different sections of the gut of arctic ground squirrels that were fed diets with different protein contents.

We obtained high quality sequence data from a subset of 38 samples (Supplementary Table S1). Pre-hibernation dietary protein content did not significantly contribute to the variation in functional composition of the gut microbiome of hibernating arctic ground squirrels for any PCA axis (Figure 2A; Supplementary Table S2). Within the gut, the cecum mucosa (*p* < 0.001), cecum lumen (*p* = 0.006), and SI mucosa (*p* = 0.04) explained 37.7% of the variation in PC1 (R^2^_ad_ = 0.377), but the SI lumen did not contribute significantly to the variation. All gut sections contributed to the variation in PC2 (*p* = 0.007-p<0.001; R^2^_ad_ = 0.386), explaining 38.6%.

Similar to the functional community PCA, we did not detect a significant contribution of pre-hibernation diet to the variation in bacterial taxonomic composition of the hibernating squirrel microbiome for any of the PCA axes (Figure 2B; Supplementary Table S2; *p* = 0.526-0.971). For PC1, gut section explained 42.5% of the variation in microbiome taxonomic composition across all samples (*p* < 0.001; R^2^_adj_ = 0.425). The cecum mucosa (*p* = 0.005), SI mucosa (*p* < 0.001), and SI lumen (*p* = 0.001) samples were all significant contributors to the variation, but not the cecum lumen. PC2 only showed a significant contribution of the cecum mucosa (*p* = 0.01; R^2^_adj_ = 0.153), explaining 15.3% of the variation in taxonomic community composition.

### Differential gene expression

**FIGURE 3:**
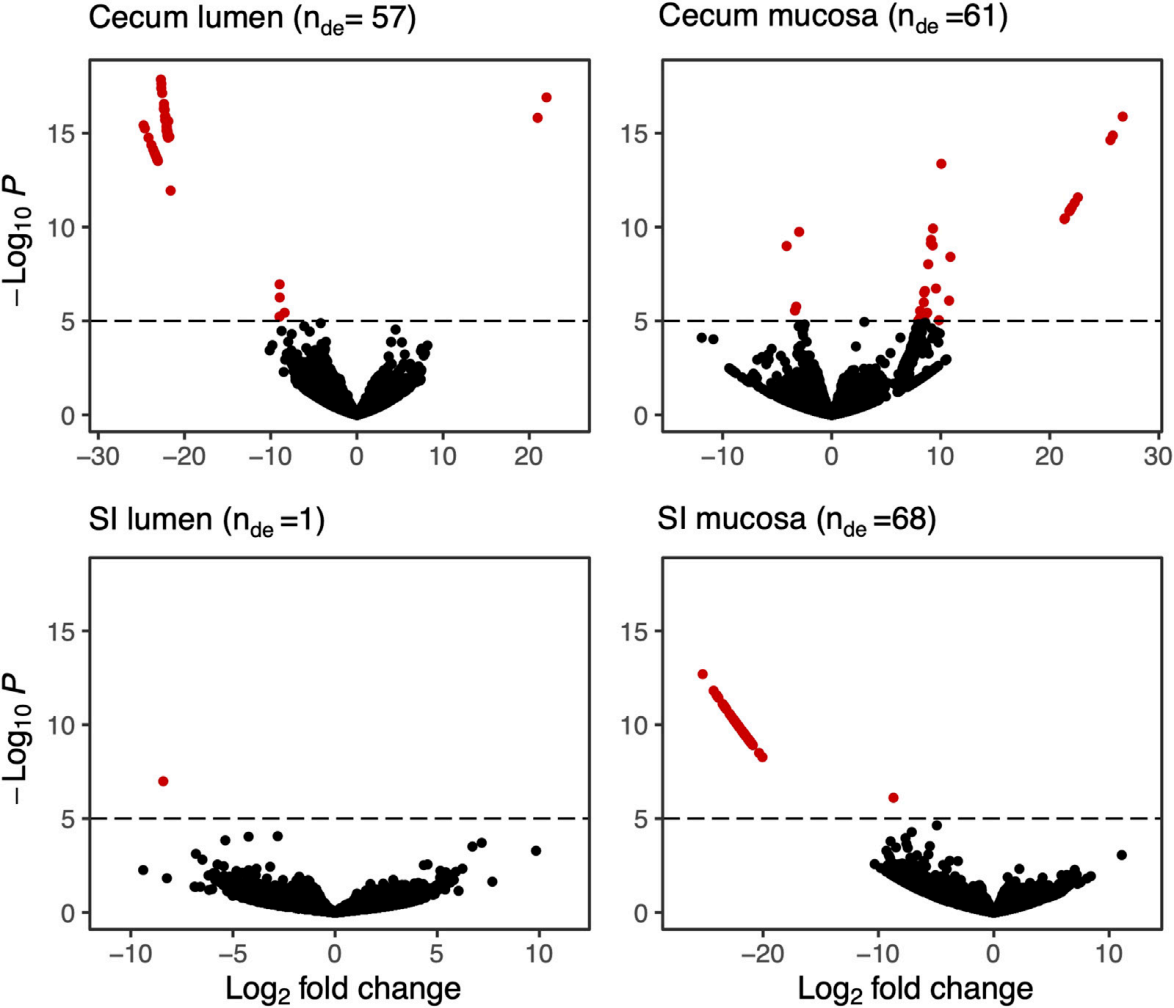
Volcano plots of differentially expressed bacterial genes in four sections of the GI tract of hibernating arctic ground squirrels fed a 9% vs. 18% protein diet during pre-hibernation. Each dot represents a KEGG ID associated with an expressed gene. Significantly differentially expressed genes are shown in red (see Supplementary Table S1 for gene descriptions) and n_DE_ shows the number of differentially expressed genes. Significance was set to 10^–6^. For a full list of differentially expressed genes see Supplementary Data Sheet 1.

We calculated differential expression for 28,197 genes detected across our samples. More differentially expressed genes were detected in the SI mucosa (*n* = 68), followed by the cecum mucosa (*n* = 61), cecum lumen (*n* = 57), and the SI lumen (*n* = 1; Figure 3), for a total of 187 (0.66%) genes expressed differentially.

In the cecum mucosa of hibernating squirrels, the expression of 20 metabolism-associated genes was significantly higher in squirrels fed 18% dietary protein compared to 9% dietary protein prior to hibernation and no squirrels fed the 9% diet showed differential expression in this pathway group (Supplementary Figure S1). In the cecum lumen the expression of 21 metabolism genes was higher in squirrels fed 18% protein prior to hibernation, while two genes (K01736: Amino Acid metabolism: Phenylalanine, tyrosine and tryptophan biosynthesis; K02302: Metabolism of cofactors and vitamins: Porphyrin metabolism) were more highly expressed in squirrels fed 9% dietary protein.

All genes that were differentially expressed in the SI mucosa of hibernating squirrels were more abundant in squirrels fed 18% dietary protein prior to hibernation. Genes encoding metabolism pathways comprised 40% of the differentially expressed genes (Supplementary Figure S2), while 14% of differentially expressed genes encoded amino acid-related pathways (Table 2). Within the metabolism pathways in the SI lumen, one gene (K07738; Protein families: genetic information processing: Transcription factors) was more highly expressed in squirrels fed 18% dietary protein prior to hibernation.

**TABLE 2:** Differentially expressed pathways within the amino acid metabolism pathway group. The diet column represents which pre-hibernation diets showed significantly higher expression of the associated Kegg ID.

	KeggID	Diet	Pathway	Enzyme
Cecum mucosa	K01736	18%	Phenylalanine, tyrosine, and tryptophan biosynthesis	Chorismate synthase
Cecum lumen	K01736	9%	Phenylalanine, tyrosine, and tryptophan biosynthesis	Chorismate synthase
	K00014	18%	Phenylalanine, tyrosine, and tryptophan biosynthesis	Shikimate dehydrogenase
	K00611	18%	Arginine biosynthesis	Ornithine carbamoyltransferase
	K00639	18%	Glycine, serine and threonine metabolism	Glycine C-acetyltransferase
	K01580	18%	Alanine, aspartate, and glutamate metabolism	Glutamate decarboxylase
	K01580	18%	beta-Alanine metabolism	Glutamate decarboxylase
	K01580	18%	Taurine and hypotaurine metabolism	Glutamate decarboxylase
SI mucosa	K01755	18%	Alanine, aspartate, and glutamate metabolism	Argininosuccinate lyase
	K15633	18%	Glycine, serine and threonine metabolism	2,3-Bisphosphoglycerate-independent phosphoglycerate mutase
	K01649	18%	Valine, leucine, and isoleucine biosynthesis	2-Isopropylmalate synthase
	K01755	18%	Arginine biosynthesis	Argininosuccinate lyase
	K00926	18%	Arginine biosynthesis	Carbamate kinase
	K13746	18%	Arginine and proline metabolism	Carboxynorspermidine synthase
	K10793	18%	Arginine and proline metabolism	D-proline reductase (dithiol)
	K11646	18%	Phenylalanine, tyrosine, and tryptophan biosynthesis	3-dehydroquinate synthase II
	K10793	18%	D-Amino acid metabolism	D-proline reductase (dithiol)
	K07160	18%	Glutathione metabolism	5-Oxoprolinase (ATP-hydrolizing) subunit A
	K01255	18%	Glutathione metabolism	Leucyl aminopeptidase

### Amino acid metabolism

**FIGURE 4:**
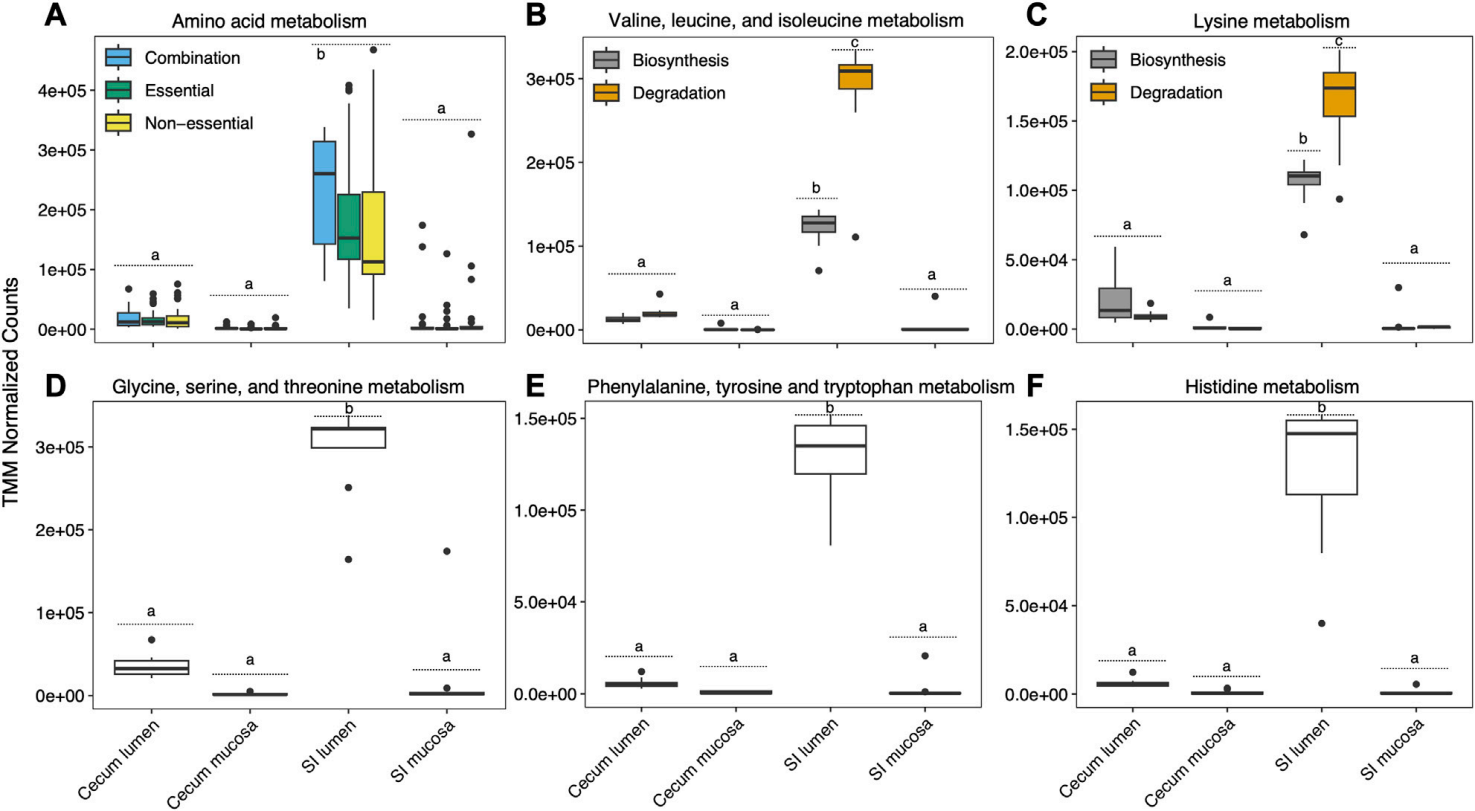
Mean normalized expression of genes associated with total amino acid metabolism and five pathways incorporating essential amino acid metabolisms. Significant differences in expression between and within sections are depicted with letters, at *p* < 0.05.

Mean normalized expression of genes associated with overall AA pathways was highest in the SI and lowest in the cecum mucosa (Figure 4A). We investigated gene expression associated with five EAA pathways. We did not find a significant effect of pre-hibernation diet on the expression of all genes associated with EAA pathways combined during hibernation in any gut section (TukeyHSD, *p* = 0.08-0.916), and we therefore did not distinguish by diet in our visualizations (Figure 4). Valine, leucine, and isoleucine biosynthesis and degradation pathway genes were almost exclusively expressed in the SI lumen (Figure 4B). Glycine, serine, and threonine metabolism, histidine metabolism, and phenylalanine, tyrosine, and tryptophan metabolism genes could not be separated into biosynthesis and degradation and were expressed predominantly in the SI lumen (Figures 4D-F). Expression of genes for these pathways was higher in the SI compared to the cecum. Lysine metabolism pathway genes were predominantly expressed in the SI lumen (Figure 4C). Genes associated with lysine biosynthesis and degradation were mainly expressed in the SI lumen, with degradation pathway genes expressed at a significantly higher level than biosynthesis pathway genes (*p* = 0.001) (Figure 4C).

Although there was no effect of diet on expression of full EAA pathways, several individual genes that are part of EAA pathways were differentially expressed, predominantly in the 18% dietary protein group (Table 2). A gene associated with the phenylalanine, tyrosine, and tryptophan biosynthesis pathways (K01736) that coded for chorismate synthase was more highly expressed in squirrels fed 18% dietary protein in the cecum lumen, but in the cecum mucosa expression of the same gene was higher in squirrels fed 9% dietary protein. In addition, genes associated with glycine, serine, and threonine metabolism (K15633), and valine, leucine, and isoleucine metabolism (K01649), were more highly expressed in the cecum lumen and SI mucosa of squirrels fed 18% dietary protein.

### Urease and the urea cycle

Mean normalized urease expression in the gut microbiome of hibernating squirrels significantly differed by gut section (Levene’s test: *p* = 0.04; Welch’s-ANOVA, F_3,29_ = 4.302, *p* = 0.025), but not by pre-hibernation dietary protein content (Levene’s test: *p* = 0.971; ANOVA, F_1,35_ = 0, *p* = 0.985). Our differential gene expression analysis did identify two genes that are part of the urea cycle and involved in arginine biosynthesis (Table 2. K00611, K01755) that were significantly more highly expressed in the cecum lumen and SI mucosa, respectively, of hibernating squirrels fed 18% dietary protein compared to 9% dietary protein prior to hibernation. No genes encoding urease biosynthesis or biodegradation were significantly differentially expressed between diet groups in any of the gut sections.

**FIGURE 5:**
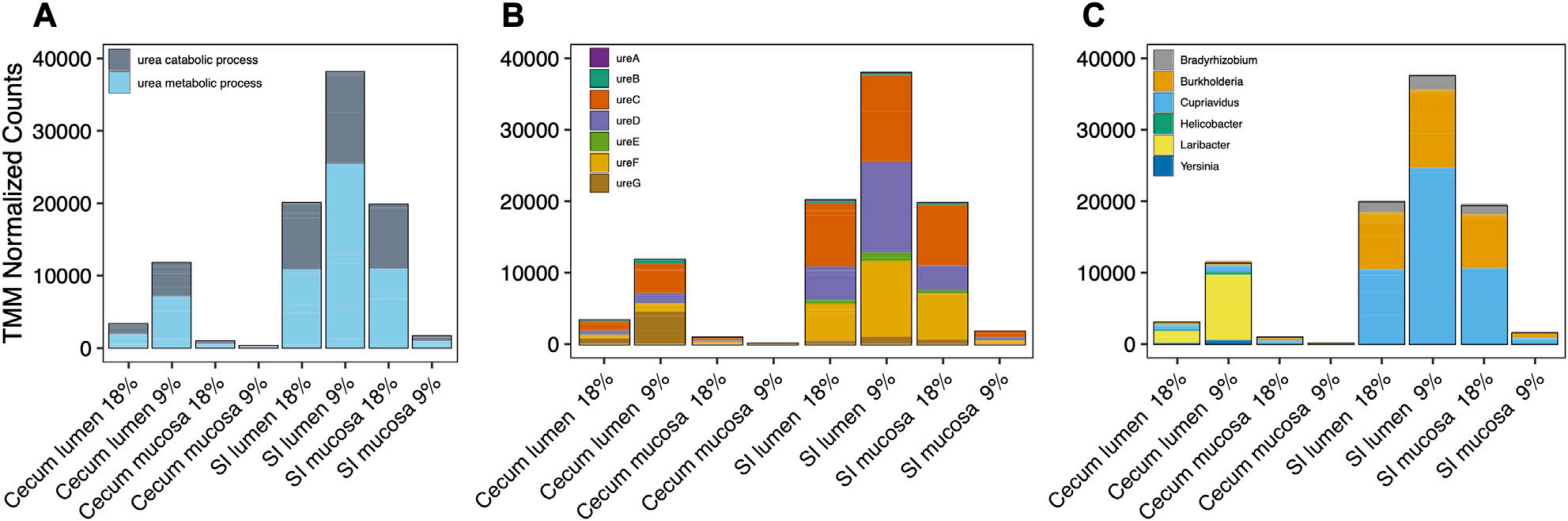
Cumulative TMM (Trimmed Mean of M-values) normalized expression depicted as **(A)** genes associated with urea catabolism:metabolism pathways, **(B)** urease genes in the urease operon, and **(C)** taxonomy of six bacterial genera that express urease genes in different gut sections of hibernating squirrels fed a 18% or 9% protein pre-hibernation diet. Genera selected were most abundant and encompassed >99% of all transcripts.

During hibernation, urea metabolism gene expression occurred predominantly in the Supplementary Figure S4; Figure 5A). There was a nearly equal abundance of expressed genes related to urea metabolism pathways in the SI mucosa and lumen microbiotas of hibernating squirrels. UreC, UreD, and UreF encompassed the majority of urease genes expressed (Figure 5B). Six bacterial genera were responsible for 99% of urease gene expression as determined by highest percentage of similarity acquired from NCBI BLAST, in order of abundance: *Cupriavidus*., *Burkholderia*, *Laribacter*, *Bradhyrizobium*, *Helicobacter*, and *Yersinia* (Figure 5C). In the SI, the most abundant genus was *Cupriavidus* (Fam. Burkholderiaceae) which included *Cupriavidus metallidurans*, *Cupriavidus necator*, and *Cupriavidus taiwanensis*. The second most abundant genus was *Burkholderia* which consisted of a single species: *Burkholderia cenocepacia*. In the cecum, *Laribacter hongkongensis* was the main contributor of urease genes.

## Discussion

We investigated the effect of pre-hibernation dietary protein content on bacterial gene expression along the gastrointestinal tract of hibernating arctic ground squirrels. Although we found differential expression of bacterial genes among gut sections, we found no effect of diet on hibernation characteristics and only a minor effect on bacterial gene expression (0.66% of genes were differentially expressed). Previous studies demonstrate not only a profound effect of hibernation on microbiota diversity and composition (Carey et al., 2013; Stevenson et al., 2014), but that the effect is modulated over time such that the community composition after ∼1 month of hibernation reflects a transition from an active season microbiome (reflecting continued relative influence of dietary carbohydrates) to a “final” hibernation microbiome (reflecting high relative influence of endogenous energy sources such as mucin) (Carey et al., 2013). Given that we sampled our squirrels after 3–4 months of hibernation it is possible that the influence of pre-hibernation diet on gut bacterial gene expression had diminished and that we would have seen a stronger signal had we sampled earlier. Our experimental timeline, in particular the timing of sample collection within the hibernation cycle, was designed for tracer metabolomics and as such may have affected our results. We allowed our squirrels to complete their last full IBA and return to torpor prior to euthanasia, thereby cycling through a body temperature (T_b_) change of < 0°C–∼35°C to < 0°C. This aspect of the experimental design enabled us to maximize the time the squirrels (and their gut microbes) were at or above a T_b_ of 0°C, and therefore maximize the time for microbial ureolysis and incorporation of liberated urea-nitrogen into microbial and host metabolites. However, because squirrels were allowed to re-enter torpor before they were euthanized, there could have been some loss of mRNA transcripts as host T_b_ decreased from euthermic conditions, thereby potentially confounding our ability to detect an effect of diet on microbial gene expression. It would be interesting for future experiments to compare samples collected during the peak of an IBA to those collected in our study.

We predicted that the expression of AA synthesis genes by the gut microbiota during hibernation would be higher in squirrels fed 9% dietary protein during pre-hibernation compared to those fed 18% dietary protein. We did not find this to be the case; rather, where there was differential expression it was consistently higher in squirrels fed 18% protein during pre-hibernation. While some of these differentially expressed bacterial genes were associated with AA synthesis pathways, others were associated with AA metabolism pathways and as such, anabolism vs. catabolism was not distinguishable. While this result was not predicted, it does confirm the potential for pre-hibernation diet to modulate activity of the gut microbiome during hibernation.

We found an effect of gut section on expression of bacterial AA and EAA metabolism genes, and within gut sections, the majority of differentially expressed genes were found in squirrels fed 18% pre-hibernation dietary protein (Table 2). This result is difficult to interpret given the few bacterial genes associated with AA metabolism pathways that were differentially expressed (19 of 28,197 total genes). Although preliminary data indicate that 9% dietary protein is sufficient to increase incorporation of microbially-liberated urea-nitrogen into host tissues in active season arctic ground squirrels (unpublished data), the relative influence of microbial activity (e.g., ureolysis or microbial metabolite synthesis) vs. host uptake of urea-nitrogen is unknown. Additionally, the 9% protein diet may not be not sufficiently depleted in nitrogen to modulate the activity of microbial AA metabolism genes 3 months into hibernation. To our knowledge there are no reports of minimum protein requirements for arctic ground squirrels, nor of the average protein content of their wild diet; thus, our dietary protein concentrations were influenced by literature in rats (e.g., Gong et al., 2010), in particular showing decreased excretion of urea when consuming low dietary protein (Younes et al., 1996). Because many of the bacterial genes that were differentially expressed are components of AA metabolism pathways (as opposed to synthesis pathways), it is possible that 18% pre-hibernation dietary protein results in higher energy availability with beneficial effects either during hibernation or in the subsequent active season. Such long-lasting effects of pre-hibernation diet have been shown in European hamsters (*Cricetus cricetus*), where a high-protein pre-hibernation diet improved hibernation with carry-over effects into the next reproductive season (Weitten et al., 2019). We did not observe differences in hibernation characteristics between diet groups and our study design did not allow for sampling of squirrels in the subsequent active season.

Within the cecum lumen, AA metabolism genes/pathways that play a role in production of neurotransmitters were differentially expressed. Two genes were associated with the shikimate pathway (K01736, K00014) for aromatic AA synthesis including tyrosine and tryptophan, which are precursors for the synthesis of a variety of neurotransmitters and hormones (Lynch and Hsiao, 2023). In addition, the gene coding for glutamate decarboxylase (K01580), which synthesizes *γ*-Aminobutyric acid (GABA) from L-glutamate, was differentially expressed in the cecum lumen in association with multiple pathways. Via the GABA shunt, bacteria may convert GABA to succinate for use in the TCA cycle (Quillin et al., 2021), suggesting the use of AAs in energy generation by the microbiota. Interestingly, GABA is also an inhibitory neurotransmitter that can modulate gut-brain responses, and GABA receptor activation is considered a logical candidate for forebrain neuronal depression during torpor (Drew et al., 2007). We did not detect higher expression of bacterial genes coding for enzymes involved in the downstream catabolism of GABA, but the potential connection between dietary protein, GABA synthesis, and hibernation presents future research avenues.

We failed to find an effect of pre-hibernation dietary protein content on urease gene expression in the microbiotas of hibernating squirrels. While urease genes are expressed in response to nitrogen limitation in some bacteria, in others expression is constitutive or upregulated in response to urea (Collins and D’Orazio, 1993). Translating pure-culture gene regulation studies to the gut environment is fraught with challenges; however, all our squirrels were injected with the same dose of urea. Our results may indicate that 9% pre-hibernation dietary protein is not sufficiently low in nitrogen to influence the community of urease-containing bacteria during hibernation. Alternatively, the profound differences in squirrel gut microbiota composition between hibernation and the active season (Carey et al., 2013; Stevenson et al., 2014), combined with the lack of dietary nutrients during hibernation may negate any influence pre-hibernation dietary protein has on UNS potential.

Expression of bacterial urease genes was higher in the SI lumen than in other gut sections. We did not account for differences in microbial numbers between the SI and the cecum, and this result is surprising given that bacterial densities in the cecum are orders of magnitude higher than in the SI (Donaldson et al., 2016). The colon is the main site of UNS humans (Stewart and Smith, 2005), and in rodents, both the colon and cecum have been identified as UNS sites (Bergen, 2015; Regan et al., 2022), with little evidence for SI involvement. We did not measure urea flux in GI tissues, and our results may reflect differences in access to urea by bacteria in the SI compared to the cecum. We are unable to ascertain whether our results translate to a greater importance of the SI as a UNS site, as this would require a more in-depth analysis of the densities of ureolytic bacteria, urea concentrations and rates of urea hydrolysis, as well as densities of urea and AA transporters in both the SI and cecum; however the results do suggest the SI may deserve renewed scrutiny.

The taxonomic composition of bacteria expressing urease genes differed markedly between the SI and cecum. The majority of urease genes in the SI microbiota were expressed by species of the genera *Cupriavidus* and *Burkholderia*. Both genera are involved in the urea cycle under varying protein conditions in plant and soil microbiomes (Sun et al., 2019), and although neither have been consistently identified as members of the human gut microbiome, they are common in the foreguts of rex rabbits (*Oryctolagus cuniculus*; Fedorov et al., 2014) and laboratory mice (Costabile et al., 2022). Neither genus was identified as a source of urease genes in the 13-lined ground squirrel (Regan et al., 2022). Given that Regan et al. (2022) investigated gene abundance as opposed to gene expression, it is possible that the expression:abundance ratio was much higher in *Cupriavidus* and B*urkholderia* compared to the high abundance genera detected by Regan et al. (2022). Alternatively, bacteria containing urease coding genes may differ between arctic ground squirrels and 13-lined ground squirrels. All *Burkholderia* represented in the SI microbiotas belonged to the same species, *B. cenocepacia*, which is urease positive (O’Grady and Sokol, 2011) and is known to upregulate urease under nitrogen-limitation in pure culture (Lardi et al., 2015). In the cecum, *Laribacter*, *Helicobacter* and *Yersinia* sp. Expressed more urease genes than any other bacteria. We found higher expression of *Laribacter* urease genes in the cecum lumen of hibernating squirrels fed 9% dietary protein during pre-hibernation compared to those fed 18% dietary protein, which may suggest a greater reliance upon urea as a nitrogen source in squirrels fed the lower protein diet.

## Conclusion

We examined the influence of pre-hibernation diet on the function of the microbiota during hibernation, hypothesizing that a low protein diet prior to hibernation would prime the microbiota to increase bacterial urease- and AA pathway gene expression, which would be carried over to hibernation. Overall, pre-hibernation dietary protein did not appear to have a strong effect on nitrogen metabolism pathways in the gut metatranscriptome of hibernating squirrels, but gut sections differed markedly in their microbial expression patterns. Although we did not find a strong effect of dietary protein on nitrogen associated pathways gene expression, our data do suggest the potential for pre-hibernation diet to modulate gut microbiota function during hibernation and analyses of metagenomic and metabolomic data sets could shed further light on this question. Future studies could address changes in microbiota function over time during hibernation, and its potential impact on hibernation characteristics and squirrel physiology.

## Data Availability

The data presented in the study are deposited in the EBI repository (https://www.ebi.ac.uk/), accession numbers ERR11706428-ERR11706470.
