# Supplementary material for: Microbial gene expression during hibernation in arctic ground squirrels: greater differences across gut sections than in response to pre-hibernation dietary protein content
Source: Front Genet. 2023 Aug 10;14:1210143. doi: 10.3389/fgene.2023.1210143 (PMC10450147; doi:10.3389/fgene.2023.1210143)
Supplement: Supplementary file 1 [file DataSheet2.pdf]

Supplemental table 1. Sample numbers successfully sequenced by gut section and dietary protein content

	9% protein	18% protein	Total
Cecum lumen	6	5	11
Cecum mucosa	4	5	9
SI lumen	4	4	8
SI mucosa	6	4	10
Total	20	18	38

Supplemental table 2. Percent of variation explained by the functional and taxonomic microbiome composition of samples collected from the arctic ground squirrel gut. PC1-4 represent the first four PCA axes which cumulatively explained the majority of variation.

	PC1	PC2	PC3	PC4
Functional composition	21.8	13.6	6.6	5.6
Taxonomic composition	29.9	15.7	15.6	9.0

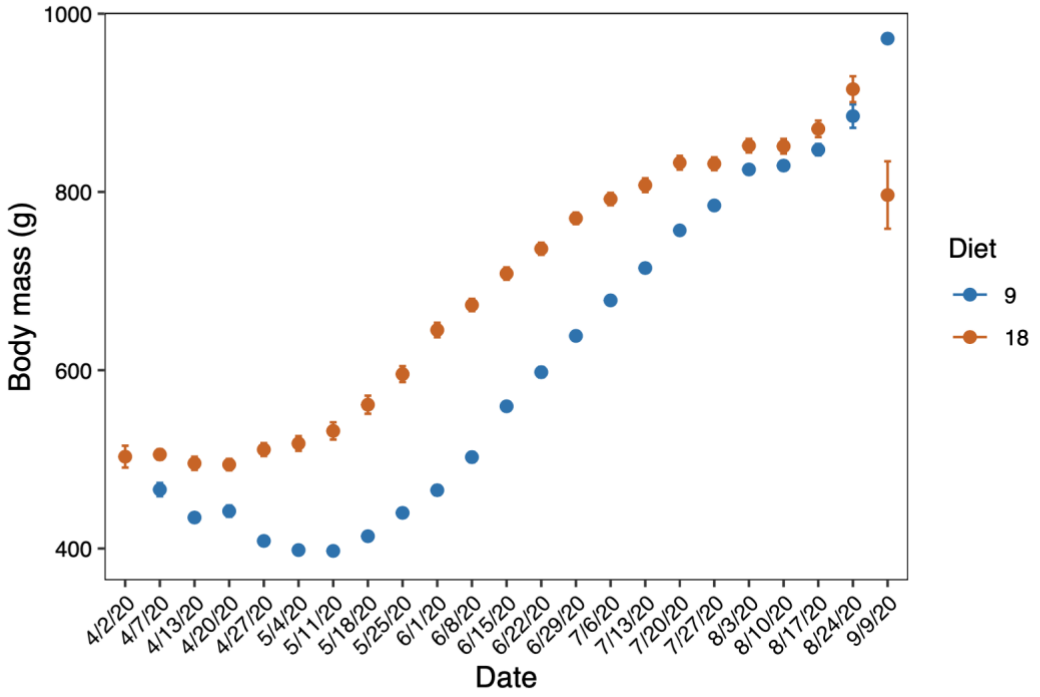

Supplementary figure 1. Weekly body mass measurements from arctic ground squirrels fed 9% or 18% dietary protein during the active season.

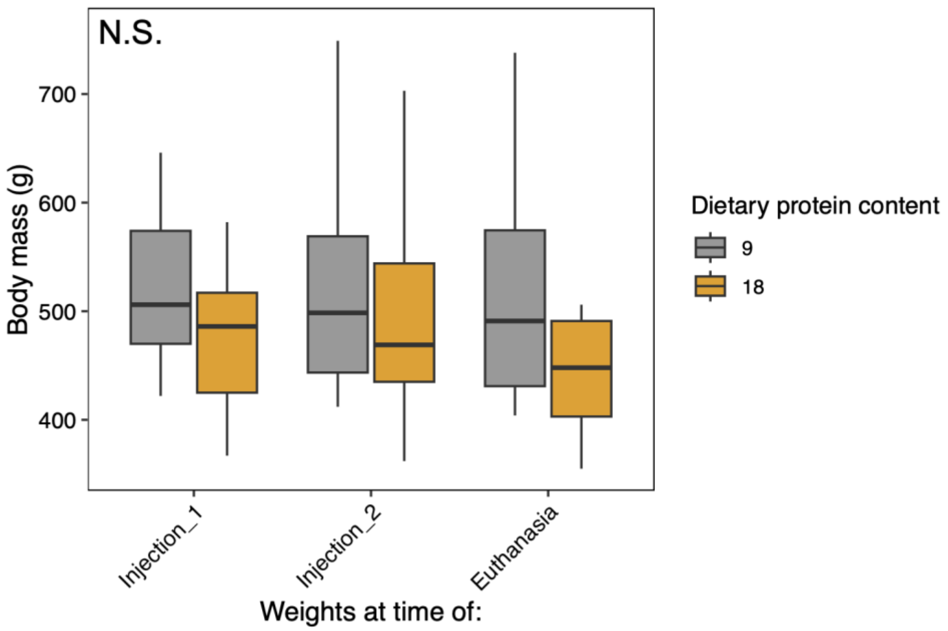

Supplementary figure 2. Body mass of arctic ground squirrels that were fed different dietary protein contents at three time points during hibernation.

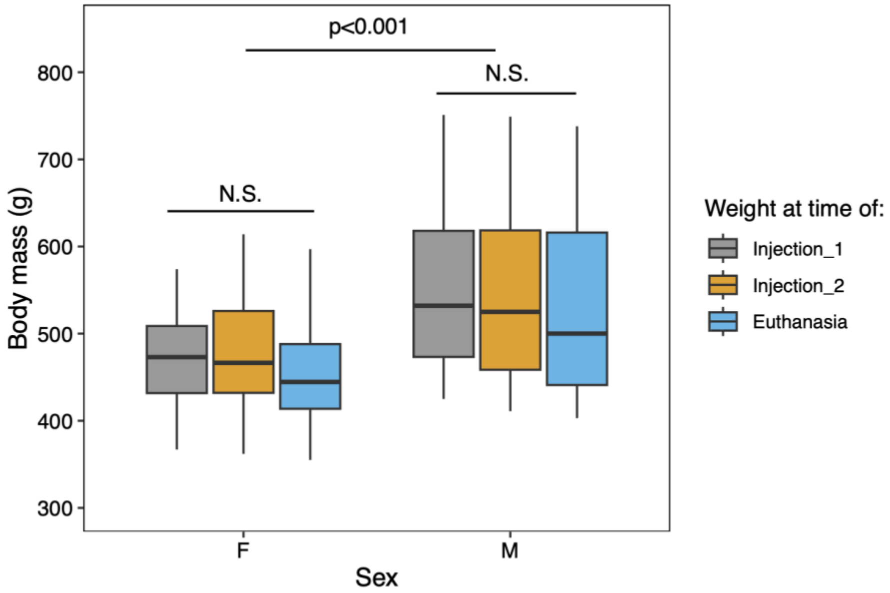

Supplemental figure 3. Body mass of female and male arctic ground squirrels at three time points during hibernation.

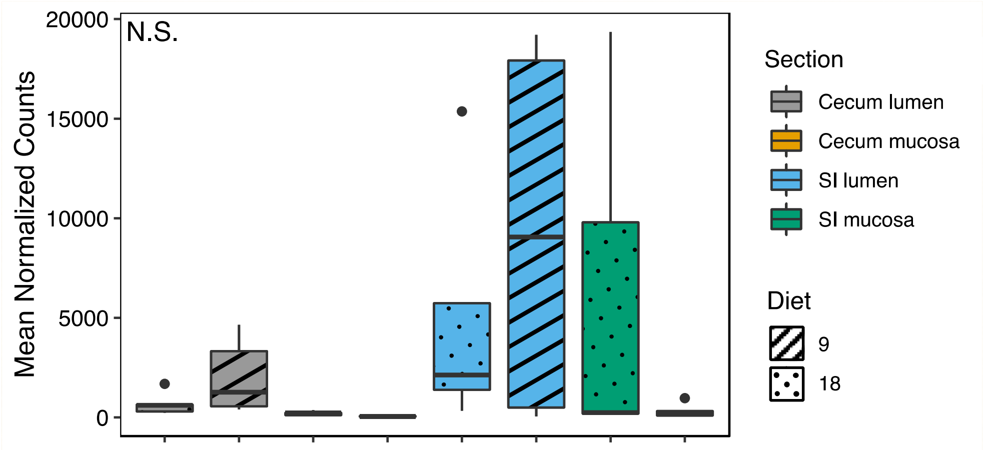

Supplemental figure 4. Mean normalized expression of urease genes in four compartments of the gastrointestinal tract of hibernating arctic ground squirrels fed a 18% or 9% protein diet during pre-hibernation.
